# Supplementary material for: RIP140 inhibits glycolysis-dependent proliferation of breast cancer cells by regulating GLUT3 expression through transcriptional crosstalk between hypoxia induced factor and p53
Source: Cell Mol Life Sci. 2022 May 3;79(5):270. doi: 10.1007/s00018-022-04277-3 (PMC9061696; doi:10.1007/s00018-022-04277-3)
Supplement: Supplementary file 1 — Supplementary file1 Supplementary Fig. 1. RIP140-deficiciency promotes cell proliferation and tumorigenesis. a, RIP140 mRNA expression relative to 28S in MCF7 and MDA-MB-436 cells transfected with control siRNA (siC) or RIP140 siRNAs (siRIP#1 used in Fig. 1a, b; siRIP#2 used in Supp Fig. 1c, d, e) (mean ± SD, n=3, ***p < 0.001). b, Cell proliferation in MCF7 and MDA-MB-436 cells transfected with control siRNA (siC) or RIP140 siRNA (siRIP#1) was measured with the xCELLigence RTCA DP instrument. The slope of the curves was extracted using the RTCA Software from the curves in Fig. 1a and 1b (mean ± SD, n=3, ***p <0.001). c, Cell proliferation assessed by 3-(4,5-dimethylthiazol-2-yl)-2,5-diphenyltetrazolium Bromide (MTT) assay in MCF7 cells transfected with control siRNA (siC) or RIP140 siRNA (siRIP#2). Values are normalized to day 1 (mean ± SD, n=3, ** p <0.01, ***p <0.001). d, Live measurements of cell proliferation were performed with the xCELLigence RTCA DP instrument in MDA-MB-436 cells transfected with control siRNA (siC) or RIP140 siRNA (siRIP#2) (mean ± SD, n=4, **p <0.01, ***p <0.001). e, Left panel: The slopes of the curves were extracted using the RTCA Software from Supplementary 1d for MDA-MB-436 and from live measurements of cell proliferation performed with the xCELLigence RTCA DP instrument in DU145 and RKO transfected with control siRNA (siC) or RIP140 siRNA (siRIP#2) (mean ± SD, n=3, *p <0.05, **p <0.01, ***p <0.001). Right panel: RIP140 mRNA expression relative to 28S in DU145 and RKO cells transfected with control siRNA (siC) or RIP140 siRNA (siRIP#2). (mean ± SD, n=3, *p <0.05, ***p <0.001). f, RIP140 mRNA expression relative to RS9 in MEFs used in the study and generated from four different breedings. MEF #1 were immortalized by the 3T3 protocol. MEF#2, #3 and #4 were transformed by the infection of SV40/H-RasV12 expressing retrovirus (left panel, mean ± SD, n=6, ***p <0.001). Immunofluorescence imaging of RIP140 protein in MEF #1. Hoechst 33342 was [file 18_2022_4277_MOESM1_ESM.pdf]

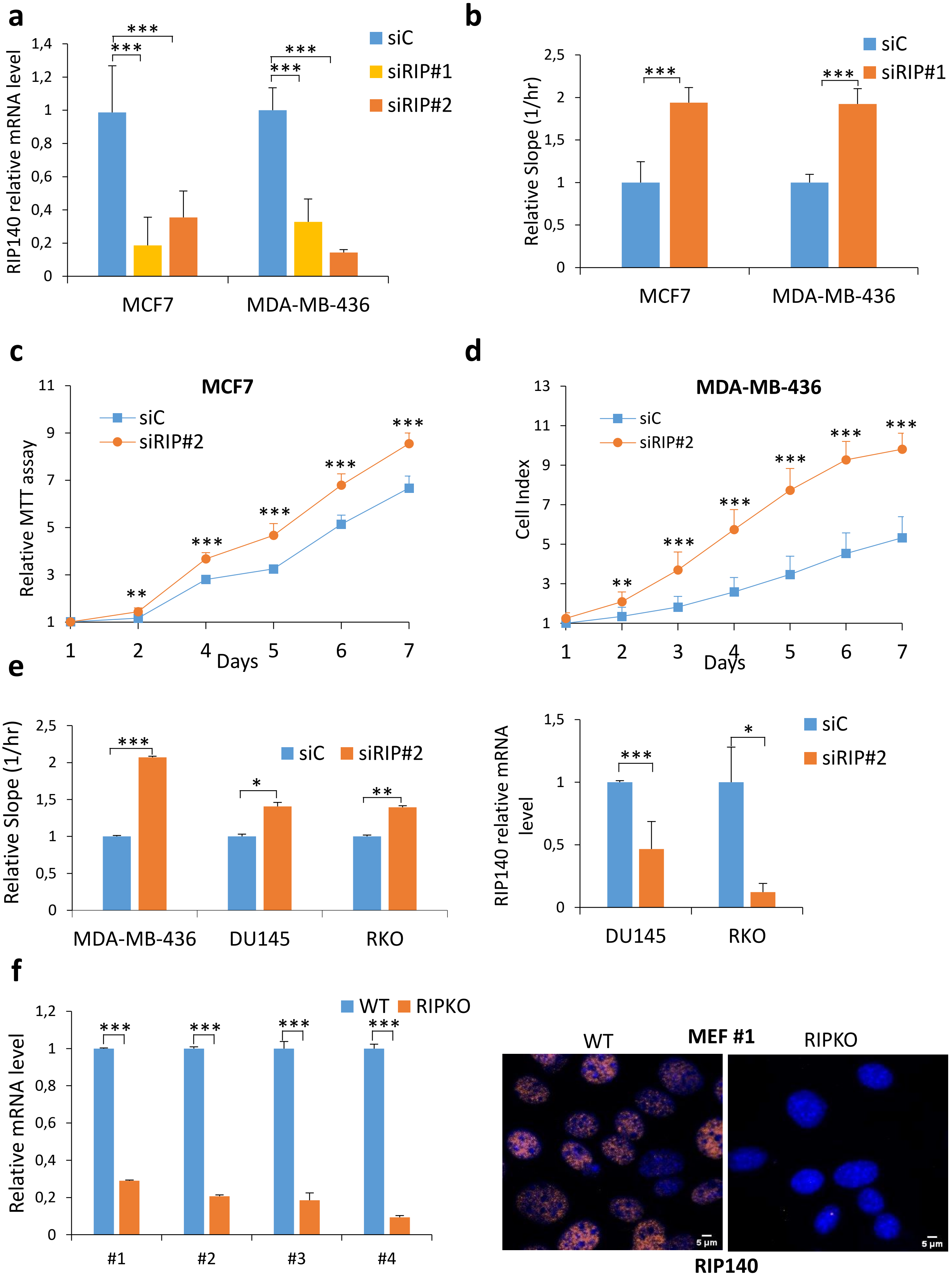

Supplementary Figure 1.

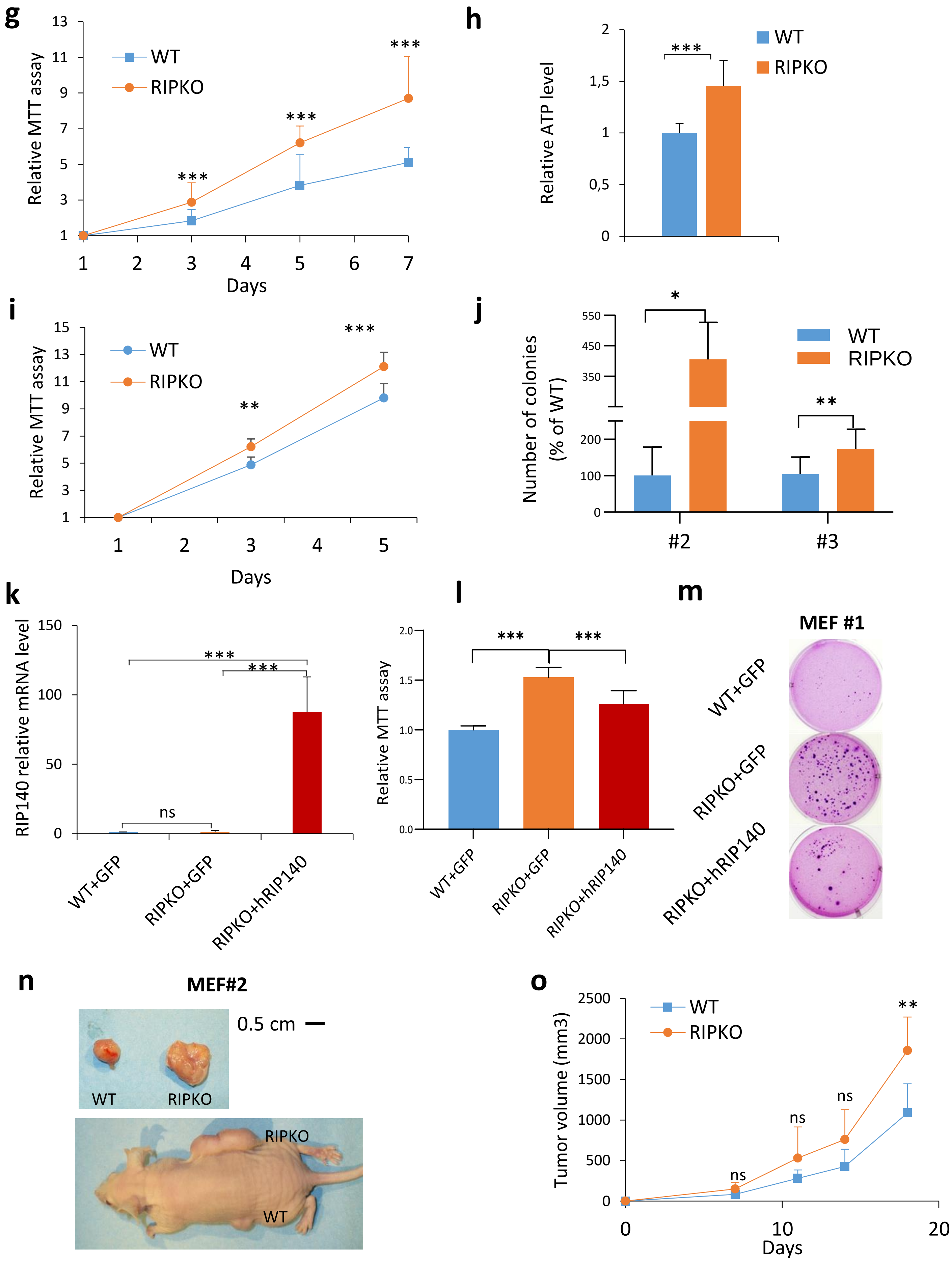

Supplementary Figure 1.

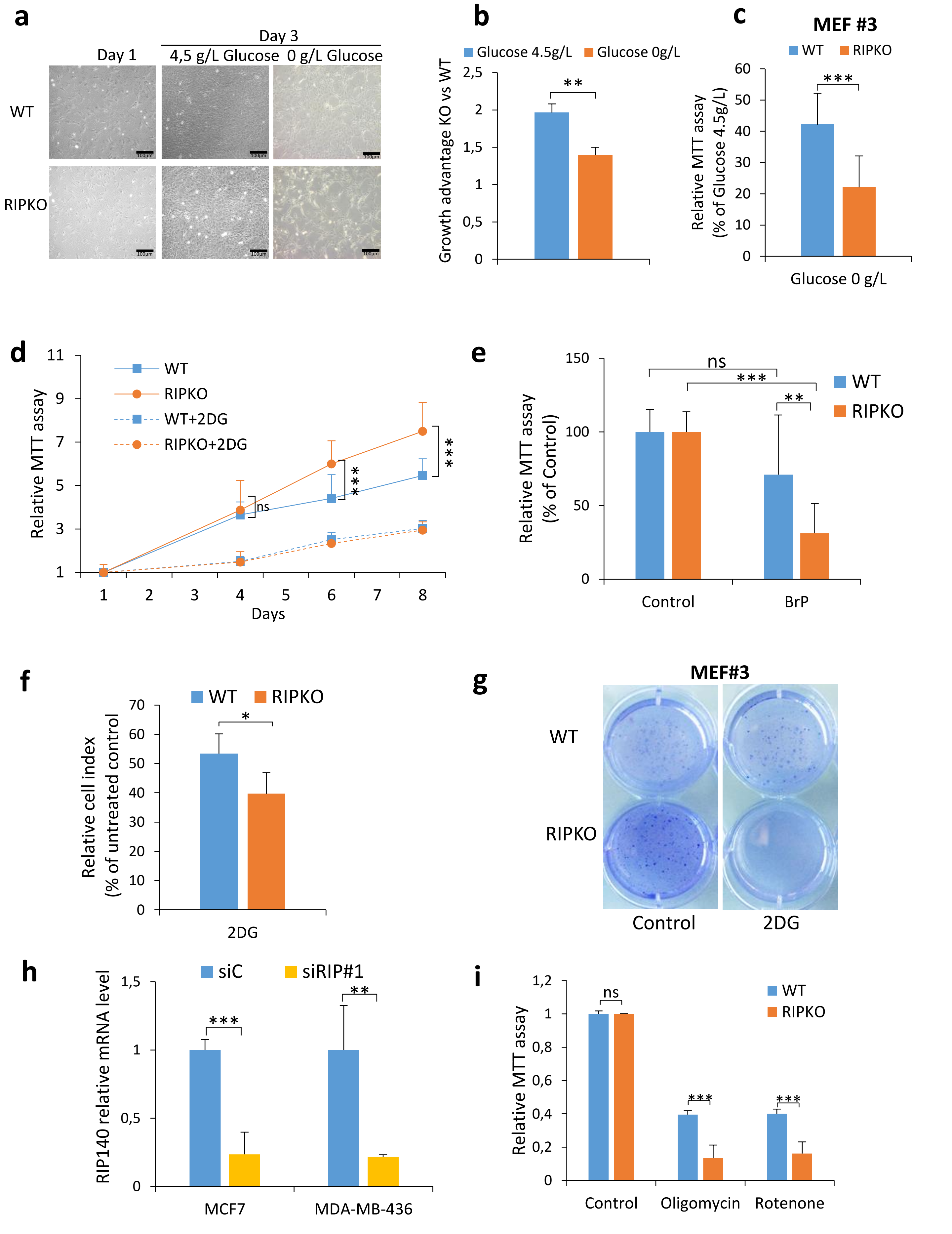

Supplementary Figure 2.

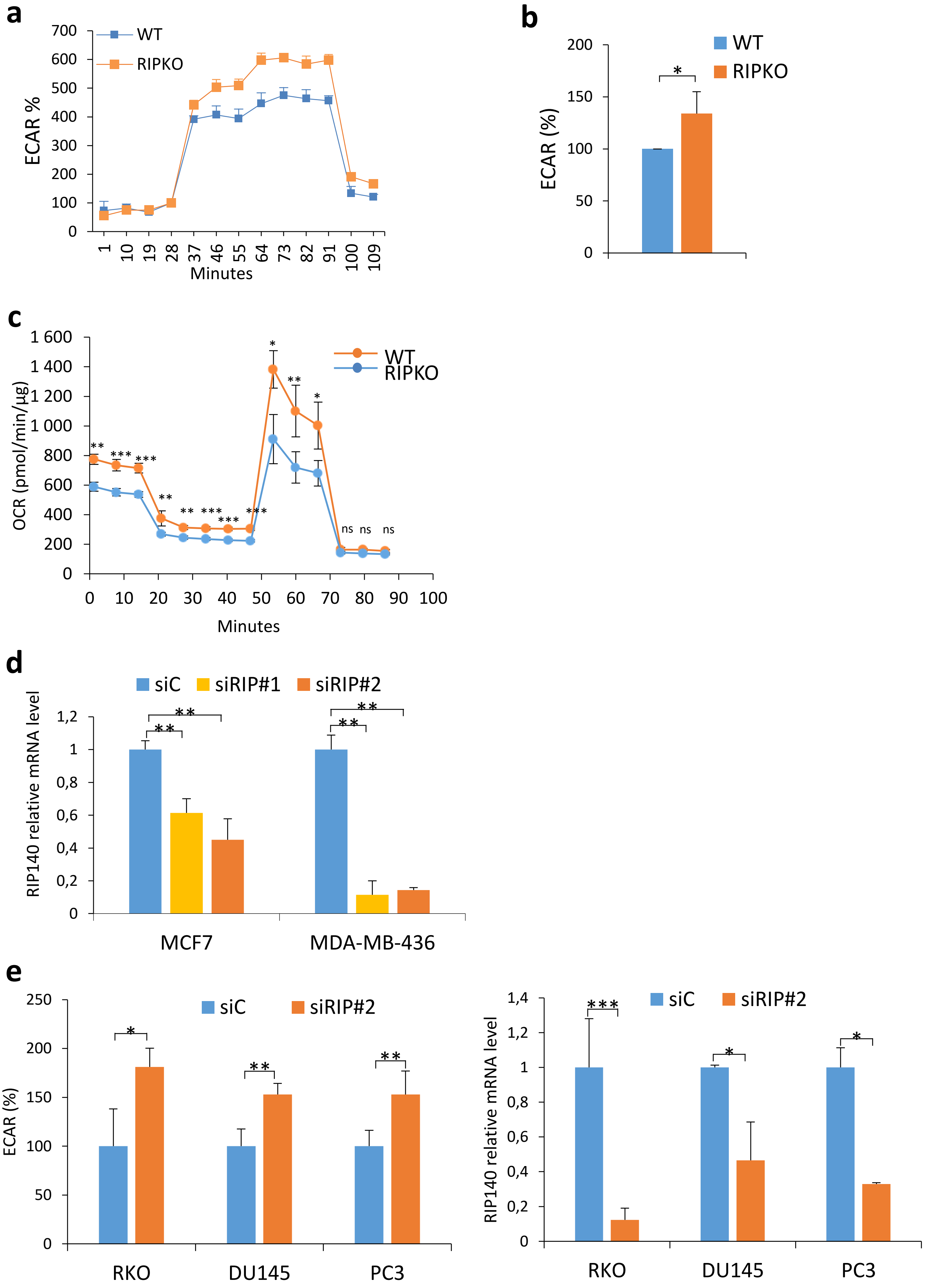

Supplementary Figure 3.

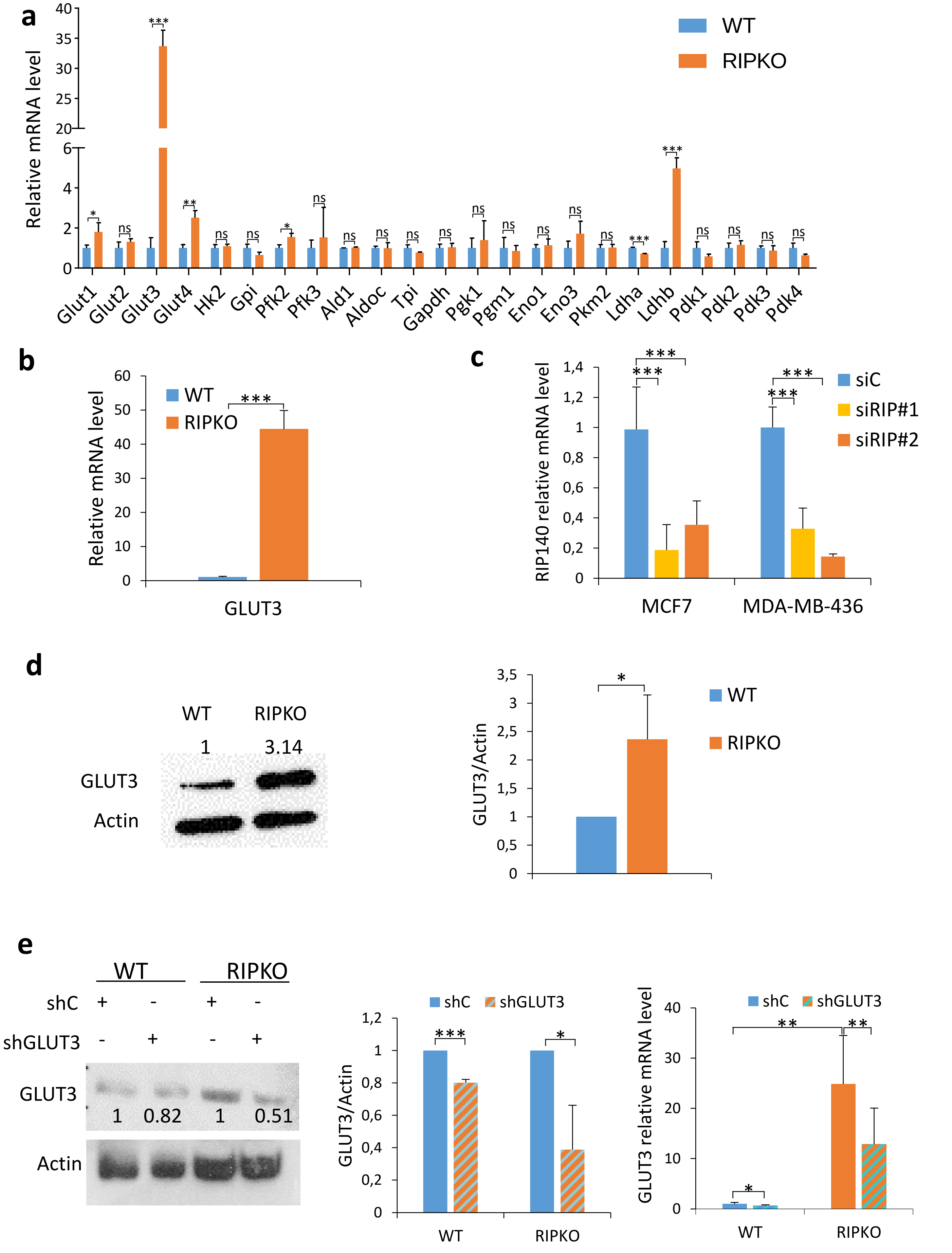

Supplementary Figure 4.

f

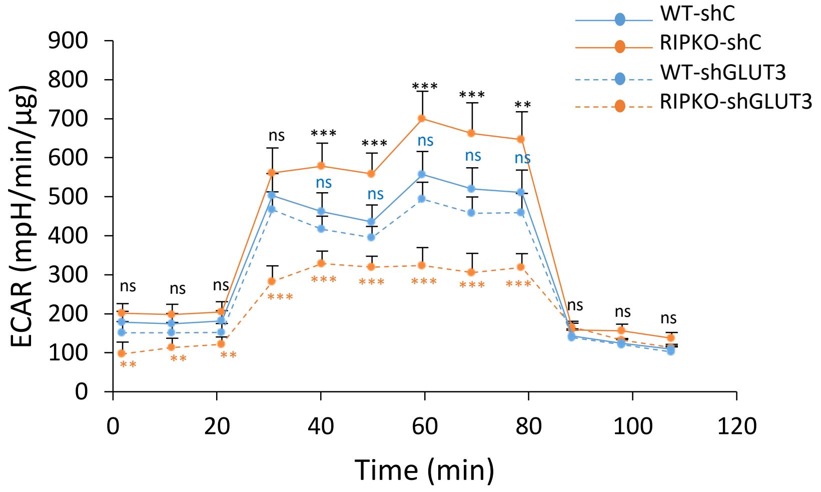

g

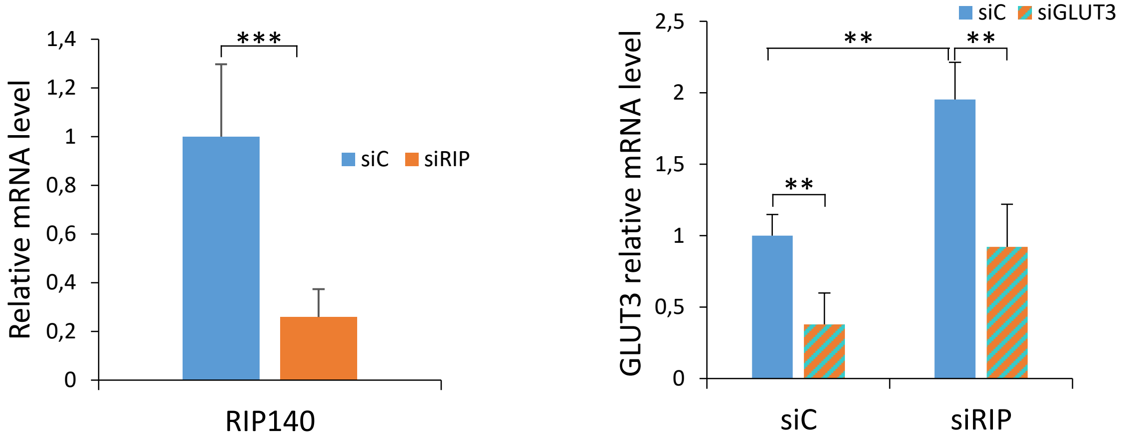

Supplementary Figure 4.

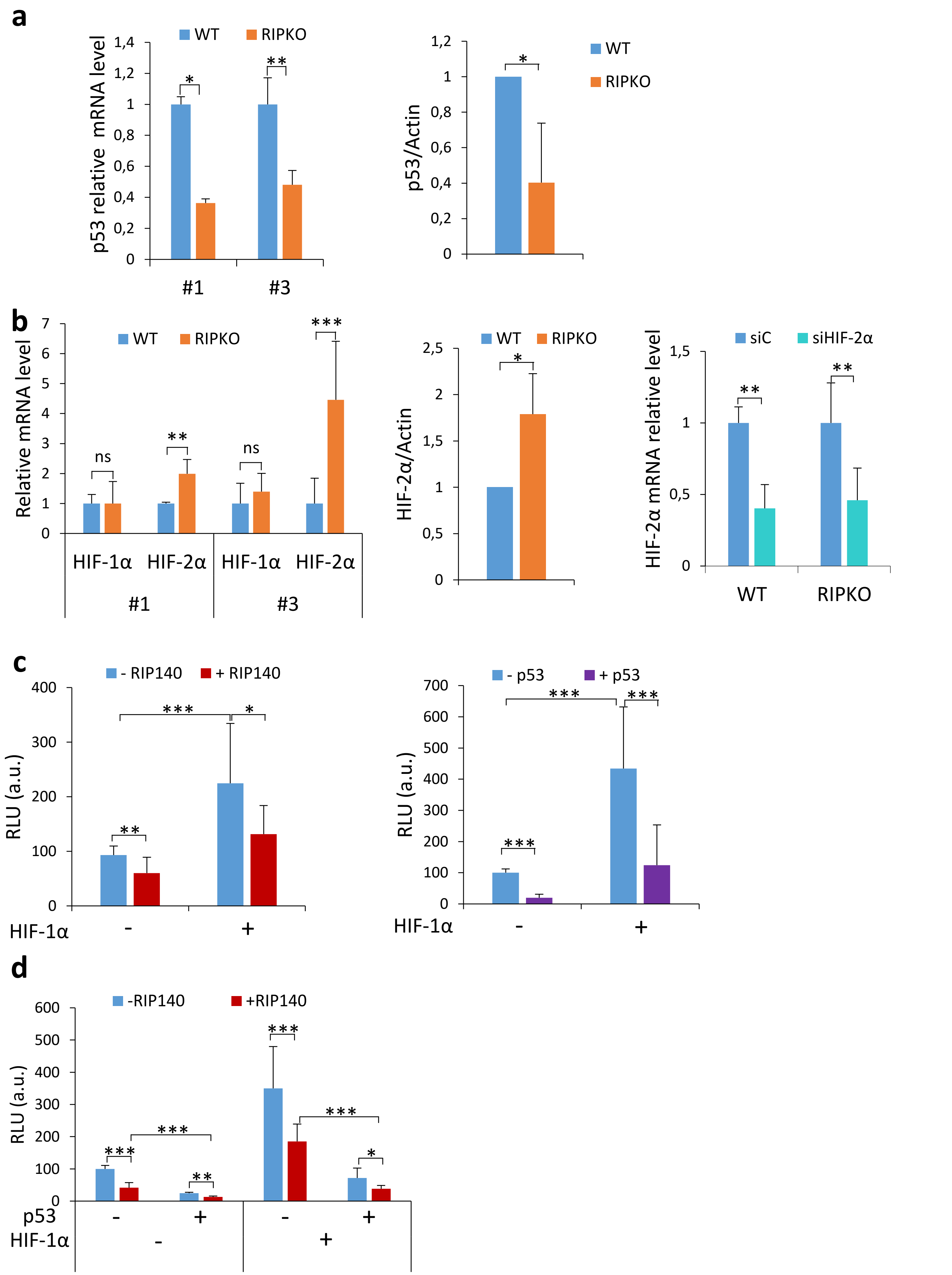

Supplementary Figure 5.

a

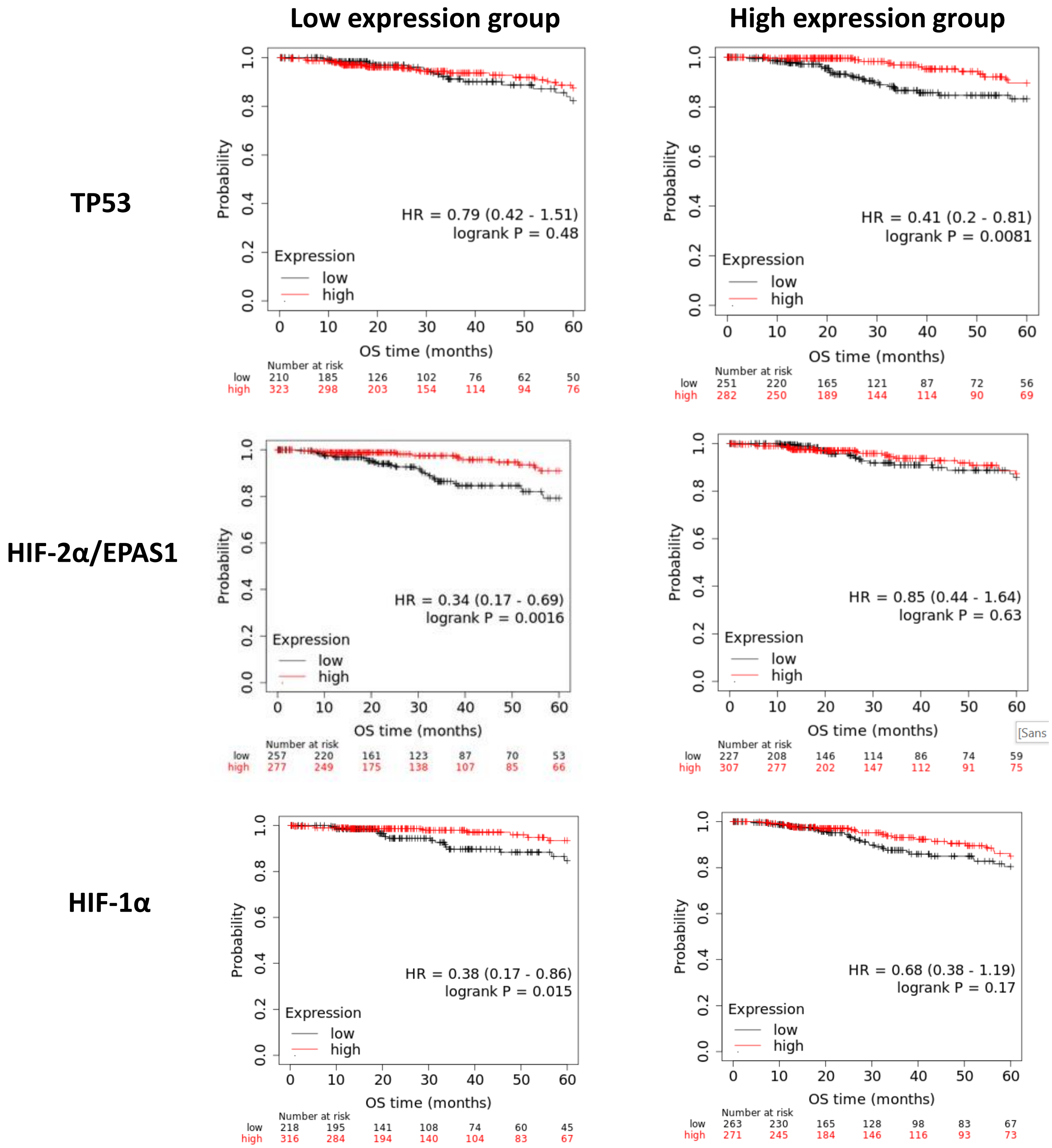

b

Tumor type	Nb of patients	Gene	Subgroup	NRIP1 cut-off	Nb of patients Low NRIP1	Nb of patients High NRIP1	HR	CI	p value
**Colon**	**450**	SLC2A3	Low	931	102	123	0.49	0.24 - 1.01	0.048*
			High	931	82	143	1.16	0.63 - 2.16	0.63
**Stomach**	**388**	SLC2A3	Low	1194	100	94	0.54	0.31 - 0.92	0.022*
			High	1194	80	114	1.15	0.76 - 1.74	0.51

Supplementary Figure 6.

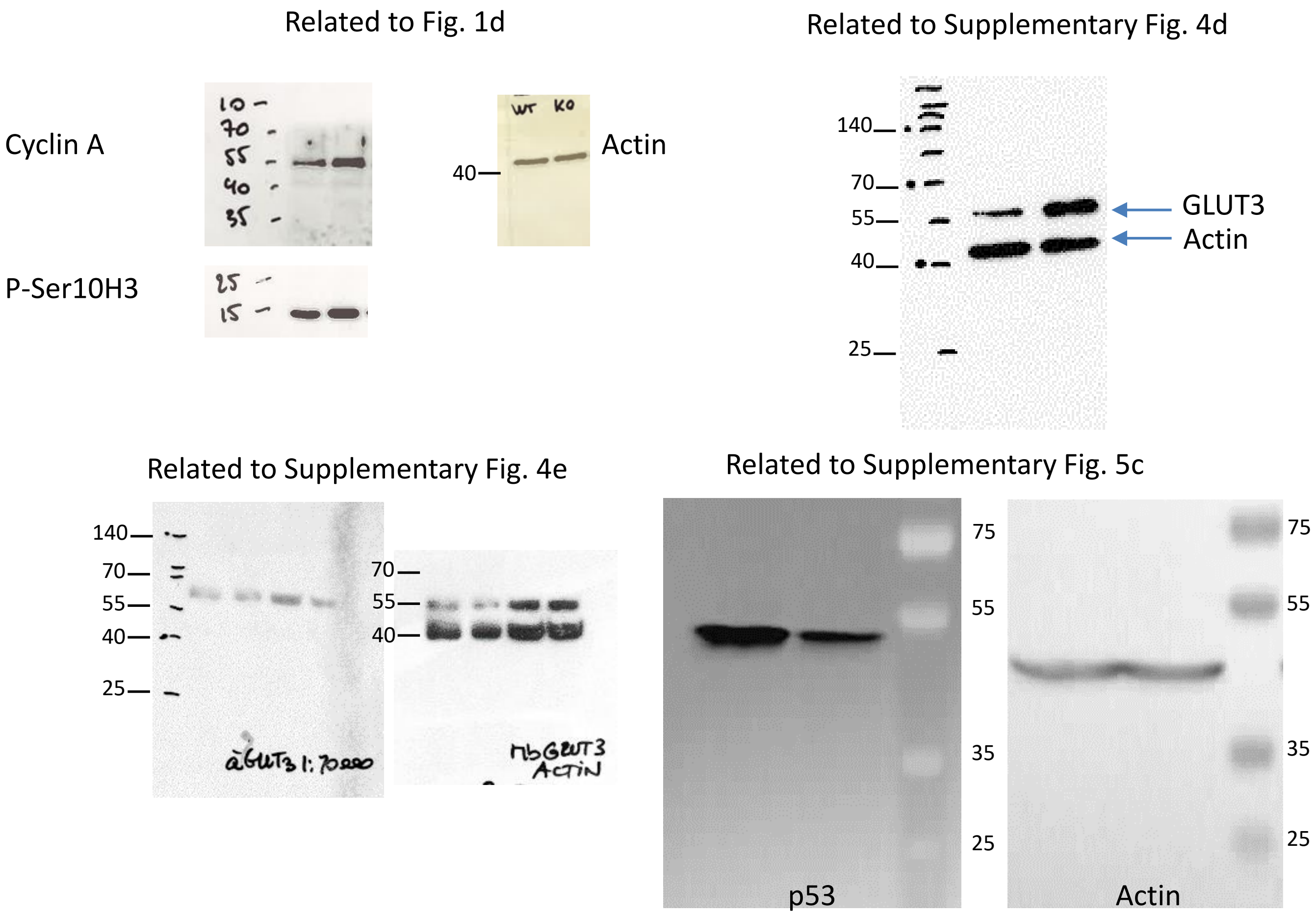

Supplementary Figure 7.

Related to Supplementary Fig. 5e

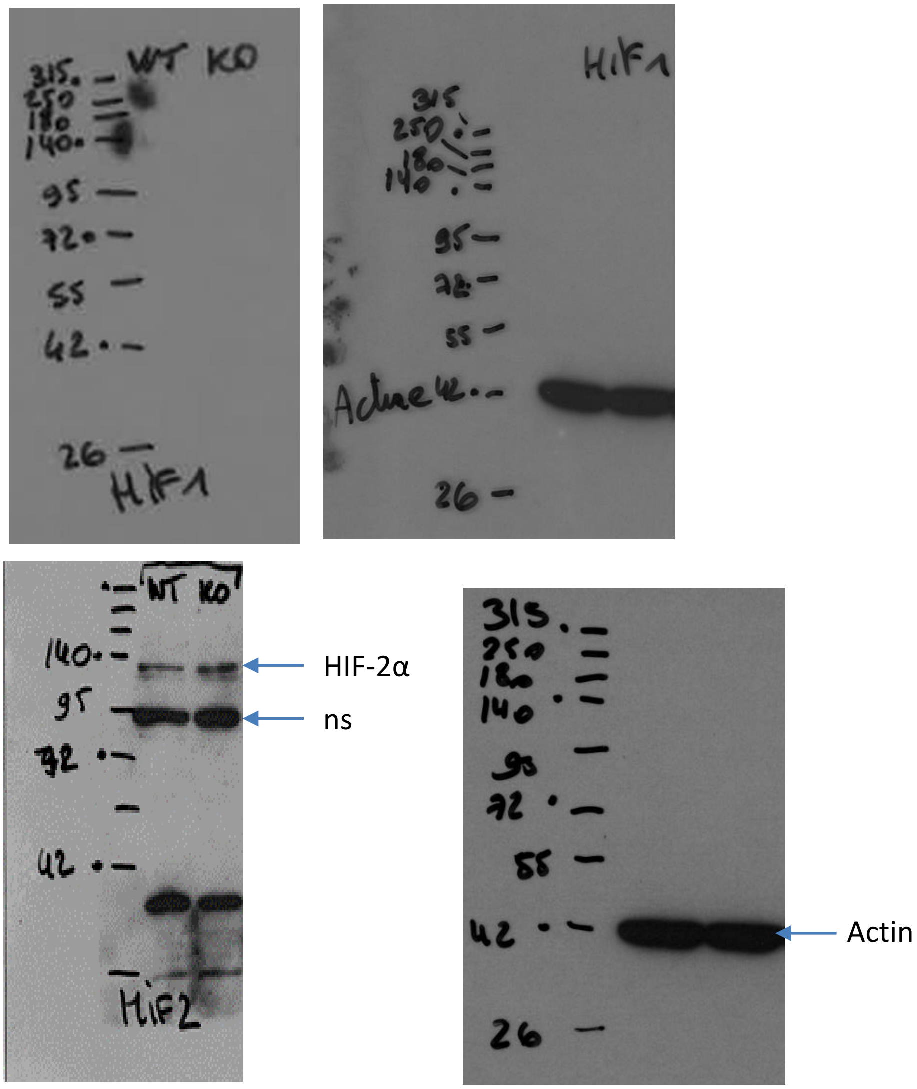

Supplementary Figure 7. Unprocessed original scans of blots.
Unprocessed images of all Western blots as indicated. Molecular size markers in kDa.
