# Supplementary material for: RIP140 inhibits glycolysis-dependent proliferation of breast cancer cells by regulating GLUT3 expression through transcriptional crosstalk between hypoxia induced factor and p53
Source: Cell Mol Life Sci. 2022 May 3;79(5):270. doi: 10.1007/s00018-022-04277-3 (PMC9061696; doi:10.1007/s00018-022-04277-3)
Supplement: Supplementary file 2 — Supplementary file2 (DOCX 44 kb) [file 18_2022_4277_MOESM2_ESM.docx]

**Table1. Murine primer sequences**

| **Name** | **Sequence** |
| --- | --- |
| ALD1-FOR | TTCGCCTACCCACCGGCGTA |
| ALD1-REV | GGTGGCAGTGCTTTCCTGGCA |
| ALDOC-FOR | ACCATATTGGGCTTGAGCAG |
| ALDOC-REV | CCTCAAACGTTGCCAGTATGT |
| ENO1-FOR | AAAGATCTCTCTGGCGTGGA |
| ENO1-REV | CTTAACGCTCTCCTCGGTGT |
| ENO3-FOR | GGACTCCAGGGGCAACCCCA |
| ENO3-REV | TGCTCGGAATCGACCCTTGGC |
| GAPDH-FOR | GTGCAGTGCCAGCCTCGTCC |
| GAPDH-REV | CAGGCGCCCAATACGGCCAA |
| GLUT1-FOR | GAGTGTGGTGGATGGGATG |
| GLUT1-REV | AACACTGGTGTCATCAACGC |
| GLUT2-FOR | GGCTAATTTCAGGACTGGTT |
| GLUT2-REV | AACCAGTCCTGAAATTAGCC |
| GLUT3-FOR | ATCGTGGCATAGATCGGTTC |
| GLUT3-REV | TCTCAGCAGCTCTCTGGGAT |
| GLUT4-FOR | CAGTGTTCCAGTCACTCGCT |
| GLUT4-REV | TTTTAAAACAAGATGCCGTCG |
| GPI1-FOR | AAAGTCCAATGGCTGACCAC |
| GPI1-REV | CACGGCCAAAGTGAAAGAGT |
| HIF1-FOR | AAACTTCAGACTCTTTGCTTCG |
| HIF1-REV | CGGCGAGAACGAGAAGAA |
| HIF2-FOR | ATC ACG GGA TTT CTC CTT CC |
| HIF2-REV | GGT TAA GGA ACC CAG GTG CT |
| HK2-FOR | GGAACCGCCTAGAAATCTCC |
| HK2-REV | GGAGCTCAACCAAAACCAAG |
| LDHA-FOR | GCAACATTCACACCACTCCA |
| LDHA-REV | TCCGTTACCTGATGGGAGAG |
| LDHB-FOR | GCTGCAGGTCCATCATCTCT |
| LDHB-REV | GTTGGACAAGTGGGTATGGC |
| PDK2-FOR | TTCCATGATGTCCAGCAGAC |
| PDK2-REV | GATCAACCTGCTTCCTGACC |
| PDK1-FOR | TTACTCAGTGGAACACCGCC |
| PDK1-REV | GTTTATCCCCCGATTCAGGT |
| PDK3-FOR | GTTAGCCAGTCGCACAGGA |
| PDK3-REV | CGTCGCCACTGTCTATCAAA |
| PDK4-FOR | TGACAGGGCTTTCTGGTCTT |
| PDK4-REV | AGTGAACACTCCTTCGGTGC |
| PFK2-FOR | AATGAGTGTTGGGGAGTTGG |
| PFK2-REV | CAGAAGACTGCAACAGCAGC |
| PFK3-FOR | CCACCATCACAATCACGGT |
| PFK3-REV | CAGAGCCGGGTACAGAAGAT |
| PG1-FOR | GTTCTTCAATGGCACCGACT |
| PG1-REV | AGTGGGACTGAGCCCAAAAT |
| PGK1-FOR | CAGCCTTGATCCTTTGGTTG |
| PGK1-REV | CTGACTTTGGACAAGCTGGA |
| PKM2-FOR | GCCGCCTGGACATTGACTC |
| PKM2-REV | CCA TGAGAGAAATTCAGCCGAG |
| p53-FOR | GAAGTCCTTTGCCCTGAAC |
| p53-REV | CTAGCAGTTTGGGCTTTCC |
| TPI1-FOR | AAAGTCGATGTAAGCGGTGG |
| TPI1-REV | GAAGTGCCTGGGAGAACTCA |

**Table 2. ChIP primer sequences**

| **Name** | **Séquence** | **Ref** |
| --- | --- | --- |
| NegCHIP-FOR | ATGGTTGCCACTGGGGATCT | [55] |
| NegCHIP-REV | TGCCAAAGCCTAGGGGAAGA. | [55] |
| GLUT3CHIP-FOR | CCCCTGAAGCAATCTTGTGATC | [55] |
| GLUT3CHIP-REV | AAAAA CCCAGGGTGGAGAGAG | [55] |

**Table 3. siRNA sequences.**

| Name | sequence | Ref |
| --- | --- | --- |
| siControl | (UAAUGUAUUGGAACGCAUA)TT |  |
| siRIP140#1 | (GAAGCGUGCUAACGAUAAA)TT |  |
| siRIP140#2 | (AUACGAAUCUUCCUGAUGU)TT |  |
| siHIF1mouse | (AAAGGACAAGUCACCACAGGA)TT | [56] |
| siHIF2mouse | (AAGUCACCAGAACUUGUGCAC )TT | [56] |
